# Automation in the High-throughput Selection of Random Combinatorial Libraries—Different Approaches for Select Applications

**DOI:** 10.3390/molecules15042478

**Published:** 2010-04-08

**Authors:** Jörn Glökler, Tatjana Schütze, Zoltán Konthur

**Affiliations:** 1 Department of Vertebrate Genomics, Max Planck Institute for Molecular Genetics, Ihnestr. 63-73, 14195 Berlin, Germany; 2 Institute for Chemistry/ Biochemistry, Free University Berlin, Thielallee 63, 14195 Berlin, Germany

**Keywords:** phage display, SELEX, automation, random combinatorial library, *in vitro* evolution

## Abstract

Automation in combination with high throughput screening methods has revolutionised molecular biology in the last two decades. Today, many combinatorial libraries as well as several systems for automation are available. Depending on scope, budget and time, a different combination of library and experimental handling might be most effective. In this review we will discuss several concepts of combinatorial libraries and provide information as what to expect from these depending on the given context.

## Introduction

**Figure 1:**
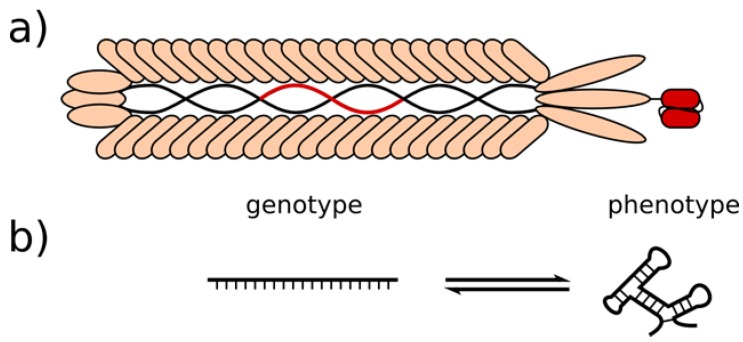
Genotype and phenotype in phage display and SELEX. (a) Schematic drawing of a filamentous phage particle. Encoding segment of single-stranded DNA genome and displayed antibody fragment are coloured in red. (b) Aptamer with interchangeable genotype and phenotype.

A new era of combinatorial libraries started in the second half of the 1980s. The invention of phage display by G.P. Smith brought about a new concept of handling large diversities by physically linking the phenotype to the encoding genotype (Figure 1a) [1,2]. This linkage allows a simultaneous physical separation of vast numbers according to certain binding properties in an iterative enrichment process. Before this invention, libraries were archived and screened by two dimensional arraying. Such a screening severely limited the library diversities to several thousands because of colony densities that can be maximally accommodated even on large Petri dishes, or simply material availability and associated costs. Applying phage display, as many as 10^13^ phage particles can be handled in a single millilitre, thus adding diversity through introduction of a third dimension.

Originally the first libraries comprised only a small diversity of peptides displayed on phage particles [3,4]. But already in 1990/91 antibody fragments [5,6] and later other molecules were displayed and much higher diversities were created, as reviewed elsewhere [7]. Also many modifications to the original format were made to allow monovalent display using phagemids [8] and even cytoplasmic proteins by using other bacterioviruses like phage λ and T7 [9,10].

Next to phage display, many variations of protein and peptide display methods based on random combinatorial libraries were developed, which are all based on the simple but effective concept of coupling the phenotype displayed with its encoding genotype. At the same time a similar, non-proteinaceous screening technology based entirely on nucleic acids was invented by Larry Gold, Andrew Ellington and colleagues in 1989 and published in 1990 [11,12]. The Systematic Evolution of Ligands by Exponential Enrichment (SELEX technology) was originally based on randomised single-stranded RNA molecules, exploiting the principle of the genotype (sequence) folding up into a more complex structure (phenotype) (Figure 1b), thus being able to bind to target molecules in a key-lock interaction. These selected binding sequences are frequently referred to as aptamers.

## Selection of Random Combinatorial Libraries

**Figure 2:**
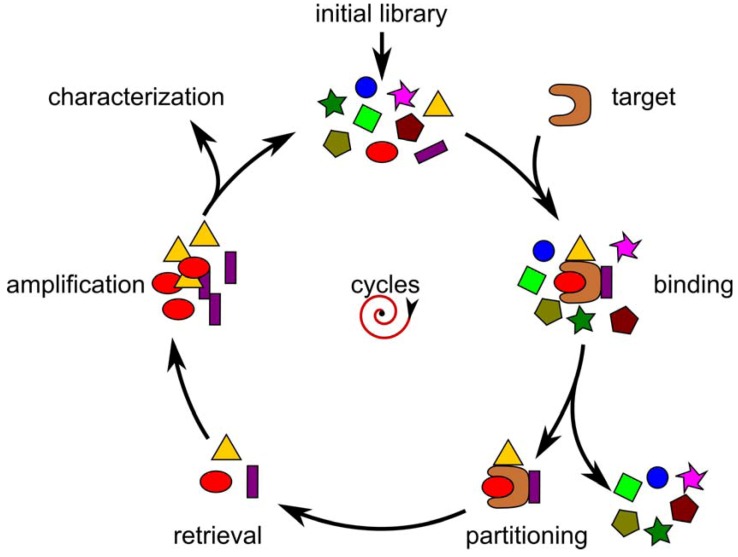
General selection scheme for random combinatorial libraries.

The screening of phage display or SELEX combinatorial libraries follows the same scheme of iterative separation and amplification (Figure 2). Because this screening process includes a certain level of mutagenesis during the amplification steps, it is also called *in vitro* evolution. The initial library is first incubated with the target molecule. The unbound variants are then washed away to retrieve the bound population in a final elution step. The eluted binders are amplified to be reintroduced in the next selection round. The screening is repeated either by a default number of selection rounds or until an enrichment of specific variants is observed.

The amount of selection rounds is dependent on the diversity of the library, affinity towards the target molecule, stringency of selection and bias for amplification. Generally it is understood that the greater the diversity of the initial library the higher the possibility of finding specific and high affinity binders. At the same time it becomes increasingly difficult to employ the tool of affinity chromatography for the separation of specific binders from the vast majority of background binders. A task that can be as challenging as finding the proverbial needle in a haystack. The solution to this problem is increasing the population of specific binders in the random pool by successive rounds of selection and amplification until either binding is observed or individual binders are sufficiently enriched for cloning and characterisation.

Over the last decades several different kinds of combinatorial libraries and their respective selection methods have been developed and an assortment of techniques is presented in Table 1. The majority is based on peptides or proteins as gene products. These offer several advantages, such as improved folding, enzymatic selection, handling of toxic proteins, or generally an instant library generation by PCR without the need of transformation [13]. 

Some newer approaches also can encode chemically synthesised compounds [14,15]. The encoded synthetic compound libraries circumvent some of the shortcomings of natural products like proteins and nucleic acids. Synthetic compounds can provide a much wider functionality by non-natural chemical groups and resistance to enzymatic degradation. However, the synthesis or translation is more time consuming and laborious than conventional enzymatic processes. So far only proof of principle selections have been published using synthetic compound libraries. Thus it remains to be seen when such new technologies will mature to become more applicable for the less experienced scientist.

**Table 1:** A selection of random combinatorial libraries technologies based on the concept of phenotype and genotype linkage.

Technology	Phenotype	Genotype	Link	Diversities	Reference	Amplification/synthesis
**Phage display**	peptide/protein	ssDNA	viral particle	10^6^–10^10^	[16]	*in vivo*/bacteria
**Bacterial display**	peptide/protein	plasmid	intracellular	10^8^–10^11^	[17]	*in vivo*/bacteria
**Yeast display**	peptide/protein	plasmid	intracellular	10^9^	[18]	*in vivo*/yeast
**Ribosome display**	peptide/protein	mRNA	complexed	10^13^	[19]	*in vitro*/cell free expression
**mRNA display**	peptide/protein	mRNA	covalent	10^13^	[20]	*in vitro*/cell free expression
***in vitro* compartimentalisation**	protein	DNA	micelle compartment	10^8^–10^11^	[21]	*in vitro*/cell free expression
**RNA SELEX**	RNA	RNA	covalent	10^15^	[22]	*in vitro*
**DNA SELEX**	DNA	DNA	covalent	10^15^	[22]	*in vitro*
**PNA display**	PNA	DNA	colavent	10^8^	[14]	*in vitro*/chemical
**DNA display**	synthetic compound	DNA	covalent	10^8^	[23]	*in vitro*/chemical

Currently, the most user-friendly systems according to our experience remain phage display and SELEX. This is primarily due to the fact that many researchers are familiar with microbial methods employed in phage display or simple nucleic acid biochemistry needed for SELEX. In the remaining sections we will hence focus on these two methods and compare them in respect to their applicability to a given scientific problem and the ease of standardisation and automation.

## Applications and Choice of Selection Technology

For obvious reasons, phage display is best for studying protein function, whereas SELEX can be used to characterise nucleic acid binding proteins or design non-coding nucleic acids with novel properties. However, several applications are common to both selection techniques. The most explored is the therapeutic potential of selected ligands. Interestingly, despite the short delay of just four years of invention of SELEX with respect to phage display, just one FDA approved drug is based on an aptamer today [24]. At the same time many therapeutic antibodies and peptides have been isolated through phage display [25]. Other applications based on selected binders include diagnostics, general biosensor design or affinity chromatography for the purification of proteins.

For each of these applications, the initial choice of selection technology and strategy is important to yield a molecule with optimal binding properties. For instance, an important consideration is the compatibility of the binder with the immune system in case of therapeutics. So far, aptamers did not elicit any adverse immune reaction [26], whereas antibodies that have not derived from completely human libraries or have not been properly humanised were shown to induce undesired side reactions [27].

When starting a new project aiming at pharmaceutical applications, it should be considered that completely synthetic molecules such as nucleic acids and peptides might be more readily characterised and formulated. This includes the synthesis according to GMP standards and coupling of chemical groups such as polyethylene glycol or lipids that increase the pharmacokinetics and pharmacodynamics [28]. Additionally, several modifications can be introduced to side chains in order to add functionality for specific interactions [29] or introduction of reactive groups allowing for photo cross-linking [30]. Another important issue is the stabilisation of these molecules against degrading enzymes [31]. Especially for aptamers one promising modification is the use of optical isomer of the natural D-ribose, called spiegelmer [32]. These spiegelmers need to be obtained by selection of normal aptamers against a mirror image of the target molecule. Such modifications and special technology are however best applied for the selection of expensive therapeutic drugs that justify the additional costs. Table 2 compares the properties of binders generated by phage display and SELEX to aid choosing the appropriate selection technology. 

**Table 2:** Feature comparison of phage display and SELEX-derived binders.

Binder	Peptide	Antibody	RNA	DNA	Spiegelmer
**Biological stability**	medium	strong	low	medium	strong
**Chemical stability**	strong	low-medium	medium	strong	as RNA or DNA
**M ultiple regeneration**	yes	no	yes	yes	yes
**Synthesis**	chemical/*in vivo*	cell culture	chemical/*in vivo*	chemical	chemical
**Adverse immune reactions**	no (size and structure dependent)	yes (needs humanisation)	no	no	no
**Synthesis cost**	low-medium	high	low-medium	low	medium-high
**Selectivity/affinity**	low-medium	high	high	medium-high	as RNA or DNA

Non-therapeutic applications are mostly less demanding on the properties because many environmental factors can be controlled. Synthesis cost, selectivity, and recycling of binders may be the more favoured features. Such properties make DNA aptamers attractive for affinity chromatography, especially for the purification of pharmaceutical products where fully synthetic ligands are preferred. Despite these advantages very little efforts have been made in this direction so far [33,34]. Besides, aptamers have acquired great attention in the field of biosensors since the target binding mechanism often includes an induced fit that can be read out by a multitude of different methods [35,36]. 

The source of libraries is an important question to be addressed. Some peptide libraries can be obtained commercially, whereas highly diverse antibody libraries (diversities exceeding 10^9^ different clones) are heavily guarded centrepieces of biotechnological companies such as MedImmune (formerly Cambridge Antibody Technologies) or Morphosys. Thus valuable antibody libraries have to be obtained by including severe restrictions or generated anew. A comprehensive list of commercial and academic phage display libraries has been published elsewhere [37]. On the other hand, SELEX libraries start with synthetic oligonucleotides that can be obtained by almost every supplier in sufficient amount and quality. However, some precautions should be taken with respect to primer design and characterisation of newly synthesised oligonucleotides [38].

Another parameter for consideration is the duration of the selections and required consumables (Table 3). Aside from the general lab equipment and expertise of the involved scientist, it should be noted that one obvious difference between phage display and SELEX is the amount of selection cycles needed to obtain specific binders. 

**Table 3:** Comparison of phage display and SELEX procedure with estimated duration of conventional protocols.

	Phage display	RNA SELEX	DNA SELEX
**Target selection**	Incubation, partitioning, retrieval, 2 hours	Incubation, partitioning, retrieval, 2 hours	Incubation, partitioning, retrieval, 2 hours
**Amplification**	reinfection, growth, superinfection, purification, 1–2 days	reverse transcription, PCR, transcription, purification, 2 days	PCR, ssDNA generation, purification, 4–6 hours
**Selection cycles**	4	10 to 15	10 to 15
**Duration of selection**	5–8 days	20–45 days	10–20 days
**Cloning**	reinfection, colony generation, picking, glycerol stocks, 2 days	vector ligation, transformation, colony generation, picking, glycerol stocks, 2 days	vector ligation, transformation, colony generation, picking, glycerol stocks, 2 days
**Characterization**	growth, superinfection, phage ELISA, 2 days	PCR, transcription, purification, FLAA, 2 days	PCR, ssDNA preparation, purification, FLAA, 2 days
**Total duration**	9–12 days	24–49 days	14–24 days
**Total cost of consumables**	low	medium-high	low

This will also influence the total duration of the selection. If any problems arise, such as amplification artefacts or contaminations during the selection cycles, it may be necessary to return to the last cycle that appeared to be correct, leading to further delays. At the same time, consumables for RNA-based SELEX are normally more expensive, because of precautions taken against ubiquitous RNases and the need of additional preparative steps including special enzymes. Furthermore, the use of chemically modified nucleotides during the selection process, which might be beneficial in regards to stability, as well as the associated use of permissive polymerases increases the costs, while simultaneously decreasing the yield of polymerase product significantly. A final consideration should include an estimation of the chances of obtaining decent binders to a given target molecule. Not all targets will be able to bind to peptides, and many will not bind to nucleic acids. Negatively charged target molecules like EGFP are particularly poor ligands for aptamers [39]. Conversely, neutral or positively charged molecules are better targets. Best estimation can be made if structural information like a crystal structure of the target is available.

## Automation of Selection and Monitoring

Automation allows the reduction of variability in the selection process, leading to a better reproducibility of the protocols. Depending on the degree of automation, time, personnel, and consumables can be reduced, whereas throughput can be increased by parallelisation. By large almost all stages in any process can be automated. In practice, the extent of laboratory automation is dependent on the scope and timeline of the project pursued, as well as the allocated budget [40]. In principle two different concepts of automation are available - full automation and unit-automation. A fully automated system refers to pipelines in which all steps of the process or assay are carried out without any human intervention [41]. In contrast, unit-automation requires human involvement in certain stages and only individual stages in the process pipeline are partially automated independent of each other [42]. In this respect, a selection pipeline has a minimum of three stages which need to be dealt with separately: the generation and immobilization of the target molecule, the binder selection process, and the screening to identify and characterize individual binders. Bearing these definitions in mind, full automation of the selection process of combinatorial libraries requires a seamless integration of interfaces and amounts to a challenging task of engineering. Quite often highly integrated systems are more prone to failure. Unit-automation preserves a highly open architecture of the pipeline allowing individual modules to be easily modified or exchanged and as a direct consequence, the pipeline can be easily extended or modified [43]. 

In a pharmaceutical or biotechnological setting the list of targets is frequently limited and much effort can be put in the quality of the molecule. In projects aiming at generation of binders towards large sets of targets the expression of protein in high-throughput is desirable. Just recently, Koehn and Hunt [44] have extensively reported on the different possibilities of high-throughput protein expression strategies using different hosts and listed commercial unit-automation solutions. Another example of a flexible, automatable expression pipeline combining a liquid handling robot with a micro-bioreactor, which allows online monitoring of the produced biomass is presented by Huber and colleagues [45].

From a binder selection perspective, next to the physicochemical parameters, major criteria for a good target are its solubility and homogeneity. Homogenous presentation is a prerequisite for any affinity enrichment process and can be achieved by directed immobilisation of the target molecule to the given selection matrix. This can be accomplished either by chemical coupling using the properties of defined residues or by using biotinylated target molecules. 

In regard of the selection procedure itself, both automation strategies have been explored. Despite some difficulties, SELEX has been fully automated with eight target selections in parallel for the first time by Ellington and co-workers [46,47]. The system consists of a pipetting robot workstation with an integrated thermal cycler, a magnetic particle separator, a vacuum filtration manifold in combination with a pipette tip carousel and an enzyme cooler, which is fully controlled by a personal benchtop computer. Similarly at Noxxon AG (Berlin), a selection robot was developed capable of performing two selections in parallel based on a different workstation in combination with units for ultrafiltration, fluorescence detection, and semi-quantitative PCR [48]. Another approach is based on a microfluidic prototype instrument, which in principle should allow the miniturisation of the SELEX procedure to be further amenable to high-throughput. However its application beyond the proof of concepts still needs to be shown [49].

**Figure 3:**
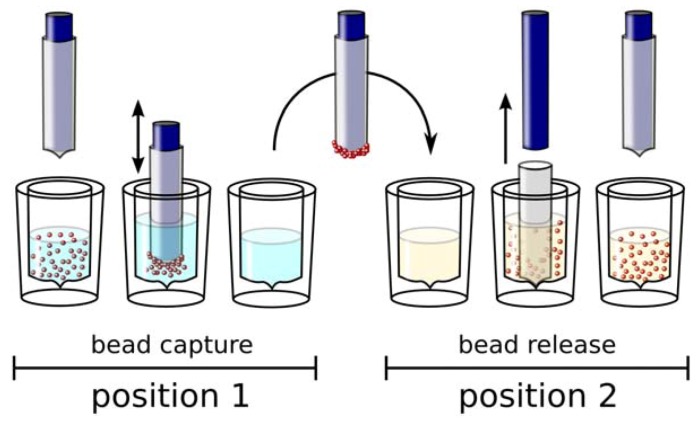
Principle of aspiration-free magnetic bead handling. Particles can be captured by a magnet covered by a plastic sheath and transferred to a new well by withdrawal of the attracting magnet from its cover (Figure taken from [55]; © 2009 BioTechniques. Used with Permission.).

Phage display includes steps involving living organisms that need to be properly monitored, hampering full automation. The initial phage display protocol has included an affinity separation step on target immobilised to immunotubes. This was later transferred to the format of microtiter plates that easily allows parallelisation and automation of the selection protocol using classical ELISA (Enzyme-Linked Immunosorbent Assay) washers [50]. However, the binding capacity of microtiter wells is limited and reduced the chance of specific interaction with libraries of large diversities. The introduction of magnetic beads has strongly improved this situation. Unlike normal affinity materials such as agarose or sepharose, magnetic particles have very little void volume and thus less of background produced by a gel filtration effect. At the same time, the small diameters in the range of 1 to 10 µm results in a favourable surface to volume ratio, which allows the presentation of target molecules in high densities [51]. Magnetic beads are easily manipulated by robotic platforms that additionally promote their use in automated selections. Today, streptavidin-coated magnetic beads that can easily be coupled with chemically or *in vivo* biotinylated target molecules, dominate in such approaches [52,53]. We have found that magnetic particle processors based on the Kingfisher® principle (Figure 3) are superior to aspiration-based platforms since contaminations due to aerosols are greatly reduced [54,55]. At the same time this handling principle is less demanding for maintenance as it involves less moving parts and exact positioning. This robotic platform has been applied first to the semi-automation of phage display [53] and more recently of SELEX [55]. All important parameters like incubation times, temperature, mixing speed, washing steps, and elution conditions can be easily programmed. Since the selection is now working in a microtiter plate format, all further steps like purification and amplification can be achieved by simple transfer between plates. Additionally, we are now using this platform for the purification of nucleic acids after the amplification step.

Automation of the selection process on its own can largely increase the throughput of selections, but also shifts the bottleneck within a selection pipeline further to the identification and evaluation side. First, the enrichment of specific binding pools can be observed by a microtiter plate based fluorescent aptamer assay (FLAA) [56] in a similar manner as ELISA is adapted to suit phage display [57]. Additionally, preliminary binding characterisation of individual clones can be achieved by such an assay. The final quantitative characterisation is normally conducted by surface plasmon resonance [50,58]. In order to monitor the flux of populations during the entire SELEX process, we have developed a new diversity assay which is based on the differential melting profiles of DNA in comparison with a synthetic standard [59]. The kinetics behind the formation of double-stranded DNA after initial thermal denaturation is dependent on the complexity and can be directly measured by generally available real time PCR equipment [60,61]. This would allow the fine-tuning of diversities for final sequencing or combination with other *in vivo* selection strategies [62].

Finally, the last step in a unit-automated selection pipeline is composed of the screening of individual binders. This demands the isolation of individual colonies, which is straight forward applying a picking robot. Cultivation of single clone in microtitre plates, expression of proteinaceous binders and performance of a binding assay of choice, such as the FLAA and ELISA, can then be performed in an automated fashion [63,64]. Further screening platforms for phage display derived binders such as protein macro- and microarrays have been described and were review by Buckler and colleagues [65].

## Conclusions and Outlook

We have successfully used a semi-automated approach for both phage display and SELEX. The chosen platform based on the magnetic particle manipulation is highly flexible and allows the selection of ligands for a multitude of downstream applications. The selection technology should be chosen carefully, because the resulting binders may prove to perform differently in the desired final application. The semi-automation has reached a considerable maturity for phage display and is now employed on a regular basis. For SELEX, we are currently evaluating the use of emulsions in the amplification steps to reduce bias and parasitic artefacts. Additionally, the use of next generation sequencing platforms circumvents the classical cloning and can reduce the amount of selection steps for the identification of binders. We expect that the current gap in the overall performance of phage display and SELEX can be reduced with the introduction of these new developments along with the presented approach to semi-automation.
